# Planktonic Growth of* Pseudomonas aeruginosa* around a Dual-Species Biofilm Supports the Growth of* Fusobacterium nucleatum* within That Biofilm

**DOI:** 10.1155/2017/3037191

**Published:** 2017-07-17

**Authors:** James C. Wang, Joehassin Cordero, Yan Sun, Mayank Aranke, Randall Wolcott, Jane A. Colmer-Hamood, Abdul N. Hamood

**Affiliations:** ^1^Department of Otolaryngology-Head and Neck Surgery, University of Cincinnati Medical Center, Cincinnati, OH, USA; ^2^Department of Otolaryngology-Head and Neck Surgery, Texas Tech University Health Sciences Center, Lubbock, TX, USA; ^3^Department of Surgery, Texas Tech University Health Sciences Center, Lubbock, TX, USA; ^4^Texas Department of State Health Services, DNA Analysis Laboratory, Austin, TX, USA; ^5^School of Medicine, Texas Tech University Health Sciences Center, Lubbock, TX, USA; ^6^Research and Testing Laboratory, Lubbock, TX, USA; ^7^Department of Medical Education, Texas Tech University Health Sciences Center, Lubbock, TX, USA; ^8^Department of Immunology and Molecular Microbiology, Texas Tech University Health Sciences Center, Lubbock, TX, USA

## Abstract

**Purpose:**

The goal of this study was to understand the potential interaction between* Pseudomonas aeruginosa *and* Fusobacterium nucleatum* within the middle ear.

**Methods:**

We examined the microbiota of ear fluid and tympanostomy tubes (TTs) obtained from patients with posttympanostomy tube otorrhea. We also examined biofilms formed by* P. aeruginosa* and* F. nucleatum*, singly or together, under aerobic or anaerobic conditions.

**Results:**

While the facultative anaerobe* P. aeruginosa* dominated the bacterial population within the ear fluid, strict anaerobes, including* F. nucleatum,* dominated bacterial populations within the TTs.* F. nucleatum *was able to grow under aerobic conditions only in the presence of* P. aeruginosa*, whose growth reduced the level of dissolved oxygen within the broth to nearly anoxic condition within 4 h after inoculation. The presence of* P. aeruginosa *allowed* F. nucleatum *to maintain its growth for 72 h within the dual-species biofilm but not within the planktonic growth. Visualization of the biofilms revealed coaggregation of* P. aeruginosa *and* F. nucleatum*.

**Conclusion:**

Extrapolation of these results suggests that, within the middle ear fluid, the growth of* P. aeruginosa *produces the anaerobic conditions required for the growth of* F. nucleatum*, both within effusion and within biofilms.

## 1. Introduction

Chronic otitis media with effusion (COME) is an inflammatory process in the middle ear accompanied by accumulation of fluid [1]. In the long term, COME may result in hearing loss leading to problems in speech or language development [2]. Surgical intervention for COME includes the insertion of tympanostomy tubes (TTs). However, placement of TTs may lead to complications including posttympanostomy tube otorrhea (PTTO) and TT occlusion [3]. While many bacterial pathogens commonly associated with PTTO are facultative anaerobes, including* Pseudomonas aeruginosa*,* Staphylococcus aureus,* and* Haemophilus influenzae *[4], recent studies suggest the presence of anaerobic pathogens such as* Fusobacterium*,* Porphyromonas*, and* Peptostreptococcus *as well [5–7]. On the surface of TTs, otopathogens form biofilms within which the bacteria are surrounded by an exopolysaccharide matrix (EPS) that protects them from the effect of antibiotics and host immune responses [8]. Bacterial cells within the biofilm vary in their metabolic activity as well as in their exposure to ambient oxygen. Bacteria within the exterior surface of the biofilm have access to oxygen and nutrients, while, within the biofilm interior, the conditions are anoxic or anaerobic [9].

*P. aeruginosa, *a Gram-negative opportunistic pathogen that produces numerous virulence factors, causes infections of many body sites, including the respiratory tract and the middle ear [10, 11].* Fusobacterium*, a strict anaerobic Gram-negative bacillus, constitutes part of the normal flora of the oropharynx [7]. Fusobacteria also produce virulence factors including lipopolysaccharide, leukocidins, neutrophil-cytotoxic factors, lipases, hemolysins, hemagglutinins, and beta-lactamase [12, 13]. Fusobacteria cause local as well as invasive infections that manifest as head and neck infections, upper respiratory tract infections, or local septic thrombophlebitis with or without bacteremia [12–15].

Metagenomic techniques, including 16S rRNA-based pyrosequencing, characterize all bacteria, fungi, and viruses, at an infection site. Using 16S rRNA-based pyrosequencing, Liu et al. [16] characterized microbiota in middle ear, adenoid, and tonsil specimens obtained from pediatric patients. Results revealed the presence of diverse bacterial communities. Pseudomonadaceae dominated the middle ear microbiota while Streptococcaceae dominated tonsil microbiota. The adenoid microbiota was dominated by multiple bacteria including Pseudomonadaceae, Streptococcaceae, Fusobacteriaceae, and Pasteurellaceae [16].

It has been postulated that, during acute otitis media (AOM), the growth of anaerobic bacteria in the nasopharynx becomes possible due to changes in normal flora and the environment [17]. Könönen et al. [17] detected many anaerobes within nasopharyngeal aspirates from children with AOM, with* F. nucleatum* and* Prevotella melaninogenica* predominating. Survival time of strict anaerobic bacteria was very short when grown under aerated conditions in the absence of aerobic and/or facultative anaerobic bacteria [18]. In a previous study of the survival of four strict anaerobic species in aerated cultures, Bradshaw et al. [19] showed that the survival time for* F. nucleatum* and* Veillonella dispar* was about five min while that of* Porphyromonas gingivali*s and* Prevotella nigrescens* was less than four min. These organisms did not produce biofilms when grown alone aerobically; however, in the presence of aerobic/facultative anaerobic bacteria, these strict anaerobic species survived and grew in both planktonic and biofilm forms [18].

In this study, we investigated whether the growth of the facultative anaerobe* P. aeruginosa* would influence the survival and growth of* F. nucleatum* under aerobic conditions, either planktonically, in a mixed biofilm, or both.

## 2. Materials and Methods

### 2.1. Collection and Processing of Patient Samples

The patient was initially examined by an otolaryngologist using otoscopy to confirm diagnosis of retained tube with or without contamination. Informed consent was obtained from the parents under guidelines of this study, which was reviewed and approved by the Texas Tech University Health Sciences Center Institutional Review Board. While the study patient was undergoing standard of care treatment for chronic PTTO, ear fluid was obtained by aspiration through the tympanostomy tubes. Mucopurulent otorrhea aspirated from the external auditory canal of each ear was placed into separate sterile specimen cups. The TTs were then carefully removed using alligator forceps and placed into separate sterile specimen cups. All samples were transported to the research laboratory within 1-2 h of collection. Upon receipt in the laboratory, samples were refrigerated at 4°C for no longer than 24 h prior to DNA isolation.

### 2.2. DNA Extraction

Each TT or the mucopurulent aspirate was suspended in 500 *μ*L RLT buffer (Qiagen, Valencia, CA) in the presence of *β*-mercaptoethanol. For complete bacterial lysis, a sterile 5 mm steel bead (Qiagen) and 500 *μ*L sterile 0.1 mm glass beads (Scientific Industries, Inc., NY) were added. The samples were briefly centrifuged, the supernatant fraction was isolated, and 200 *μ*L of 100% ethanol was added to a 350-*μ*L aliquot of the supernatant. This mixture was then added to a DNA spin column and the DNA was recovered using QIAamp DNA Mini Kit (Qiagen) according to the manufacturer's recommendation. The DNA was eluted from the column with 30 *μ*L water and quantified using a NanoDrop spectrophotometer (Nyxor Biotech, Paris).

### 2.3. Amplification and Sequencing of Eubacterial 16S rRNA Genes

To amplify approximately 500 bp of the 16S rRNA genes, we used the 16S universal Eubacterial primers 28F (5′-GAGTTTGATCNTGGCTCAG-3′) and 519R (5′-GTNTTACNGCGGCKGCTG-3′) [20]. PCR was conducted using HotStarTaq Plus Master Mix Kit (Qiagen) under the following conditions: 95°C for 5 min followed by 35 cycles of 95°C for 30 s, 54°C for 40 s, and 72°C for 1 min, with a final elongation step at 72°C for 10 min. Diffinity Rapid Tip (Diffinity Genomics, West Henrietta, NY) was used to clean the PCR products. PCR products were pooled and small fragments were removed by using Agencourt Ampure Beads (Beckman Coulter, CA).

Bacterial tag-encoded FLX-Titanium amplicon pyrosequencing was done at the Research and Testing Laboratories, Lubbock, TX, as previously described (Roche, Nutley, NJ) [21]. DNA fragment sizes and concentration were accurately measured using DNA chips under a Bio-Rad Experion Automated Electrophoresis Station (Bio-Rad Laboratories, CA) and a GloMax-Multi Jr Detection System (Promega, Madison, WI). In a sample of double-stranded DNA, 7.2 million molecules, with an average size of 550 bp, were combined with 7.2 million DNA capture beads and then amplified by emulsion PCR using the same primer set with added** linker A** and 8-bp barcode sequences at the 5′-end of 28F (primer 28F-A 5′-**CCATCTCATCCCTGCGTGTCTCCGACTCAG**-barcode-GAGTTTGATCNTGGCTCAG-3′) and biotin and the** linker B** sequence added to the 5′-end of 519R (primer 519R-B 5′-Biotin-**CCTATCCCCTGTGTGCCTTGGCAGTCTCAG-**GTNTTACNGCGGCKGCTG-3′) [21]. Following bead recovery and enrichment, the bead-attached DNAs were denatured with NaOH and annealed with sequencing primers (Roche, Indianapolis, IN). Using the Genome Sequencer FLX System (Roche), a four-region 454 sequencing run was performed on a GS PicoTiterPlate. Forty tags were used on each quarter region of the plate.

### 2.4. Bacterial Strains and General Growth Conditions

The study was conducted using the prototrophic* P. aeruginosa* strain PAO1 that was originally isolated from an infected wound and the* F. nucleatum* strain VPI 4355 isolated from a cervicofacial lesion. The VPI 4355 strain was purchased from the American Type Culture Collection (Manassas, VA). For general growth, the strains were grown aerobically (18.6% ambient oxygen) or anaerobically (0% oxygen) at 37°C for up to 96 h in 37°C in brain heart infusion (BHI) broth or on BHI agar plates (CRITERION; Hardy Diagnostics, Santa Maria, CA). Biofilms were developed on 6-mm cellulose disks (Becton Dickinson and Company, Sparks, MD) in 15-mL culture tubes containing 5 mL of BHI broth. Previous studies suggested that the facultative bacteria initially grow in the middle ear and provide support for the growth of anaerobic bacteria, which arrive later as the oxygen level is reduced [22]. Therefore, to mimic in vivo conditions, strains were inoculated individually or together (coculture) at 10^6^ colony forming units (CFU) for PAO1 and 10^2^ CFU for VPI 4355.

### 2.5. Quantitative Analysis of Planktonic Cultures and Biofilms

At specific intervals depending on the experiment, the medium (planktonic culture) was removed from the tubes and serial 10-fold dilutions of the planktonic cultures were prepared. Ten *μ*L aliquots of each dilution were spotted on BHI agar plates and the plates were incubated at 37°C for 14–16 h and the CFU were counted.

The disks were removed from the culture tubes, rinsed with 1x phosphate buffered saline (PBS) to remove any planktonic cells, and transferred to 1.5 mL microcentrifuge tubes containing 1 mL of PBS. After vigorous vortexing (three times for 2-3 min each time) to mechanically disrupt the biofilms and detach the bacteria from the disks, the cell suspension was serially diluted 10-fold in PBS.

The CFU/mL (planktonic cultures) or CFU/disk (biofilms) were determined using the following formula: CFU counted × dilution factor × 100.

### 2.6. Coculture of* P. aeruginosa* and* F. nucleatum*

#### 2.6.1. To Determine If* P. aeruginosa* Supports the Growth of* F. nucleatum*

Culture tubes containing 5 mL of BHI broth and a cellulose disk were inoculated with ~10^6^ CFU of* P. aeruginosa* and 10^2^ CFU of* F. nucleatum* and incubated aerobically at 37°C for 8, 24, 48, or 72 h. The disks were then removed, and the biofilms were analyzed by determining the CFU/disk as described above. The CFU/mL of* P. aeruginosa* and/or* F. nucleatum* planktonic growth was also determined.

#### 2.6.2. To Determine Whether* F. nucleatum* Continues to Grow over a Prolonged Period in a Dual-Species* P. aeruginosa*/*F. nucleatum* Biofilm

Tubes containing BHI broth and a cellulose disk were inoculated with* P. aeruginosa *and* F. nucleatum *as described above and incubated aerobically at 37°C for 96 h. The disks were then transferred into fresh BHI broth and the incubation continued for an additional 72 h under the same conditions. Cellulose disks were then removed and the biofilms were analyzed as described above.

#### 2.6.3. To Determine If Exposure of* P. aeruginosa*/*F. nucleatum* Biofilm to Ambient Oxygen Kills* F. nucleatum* within the Biofilm

The* P. aeruginosa/F. nucleatum *biofilms were developed as described above. After 24 h of incubation at 37°C, the disks were then divided into two sets. In one set, the disks were placed on the top of BHI agar plates and the plates were incubated aerobically for 48 h at 37°C. In the other set, the disks were transferred to a fresh tube of BHI broth and incubated aerobically for 48 h at 37°C. The disks were then removed from either the agar plate or the tube and the biofilms were analyzed.

#### 2.6.4. To Measure Dissolved O_2_ (DO_2_) in the Culture Medium

Cultures of* P. aeruginosa*,* F. nucleatum*, or both were prepared in 50-mL conical tubes containing 15 mL of BHI broth as described above. The tubes were incubated aerobically at 37°C and the level of DO_2_ (mg/L) was measured at 1, 2, 4, 8, and 24 h using a Heavy Duty Dissolved Oxygen Meter (Extech Instruments, Nashua, NH).

### 2.7. Fluorescence In Situ Hybridization (FISH)

Single- or dual-species biofilms were developed as described above and the disks were stained using FISH as previously described with slight modifications [23]. Briefly, the disks were placed in a hybridization buffer (5 M NaCl [18% v/v]/1 M Tris/HCl, pH 7.2 [2% v/v]/10% SDS [0.1%]/formamide [20% v/v] in distilled H_2_O). Fluorescent labeled oligonucleotide probes were then added at a concentration of 50 ng/*μ*L. Hybridization was conducted in microcentrifuge tubes at 46°C for 5 h. Following hybridization, disks were incubated twice for 15 min at 46°C in wash buffer (5 M NaCl [18% v/v]/1 M Tris/HCl, pH 7.2 [2% v/v]/10% SDS [0.1%]). After washing, the disks were examined by confocal laser scanning microscopy (CLSM) as previously described [23]. Oligonucleotide probes (*P. aeruginosa, *5′-/5Cy3/GCTGGCCTAGCCTCCC-3′;* F. nucleatum*, 5′-/5Cy5/CGCAATACAGAGTTGAGCCCTGC-3′) used in this study were synthesized commercially (Integrated DNA Technologies; Coralville, IA). Excitation was carried out at 546 nm for Cy3 and 633 nm for Cy5. Fluorescence emission of the probes was measured at 493–538 nm (Cy3) and 550–592 nm (Cy5).

## 3. Results

### 3.1. Microbiota of the Ear Fluid and TT from the Patient

**Figure 1:**
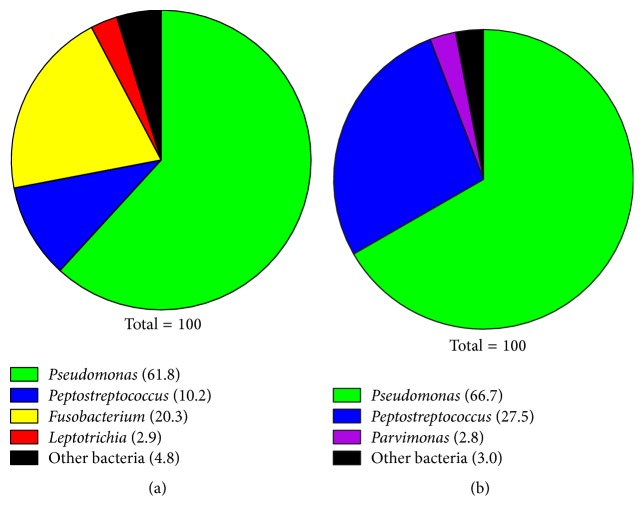
Distribution of major bacterial genera found within the ear fluid samples by deep parallel pyrosequencing. Numbers indicate the percentage of each genus present in fluid from (a) right ear and (b) left ear.* Pseudomonas *and* Streptococcus *are facultative anaerobes;* Peptostreptococcus, Fusobacterium, Leptotrichia*, and* Parvimonas *are obligate anaerobes. “Other bacteria” represent genera present at less than 2.5% each. The identities and relative abundance of these organisms are provided in Table 2.

Pyrosequencing analysis revealed that the predominant genus present in the right and left ear fluid samples was the facultative anaerobe* P. aeruginosa* (62% and 67%, resp.) (Figure 1). However, the second most predominate genera were strict anaerobes; in the right ear fluid, genus* Fusobacterium* constituted 20% and genus* Peptostreptococcus* constituted 10% of the flora (Figure 1(a)), while, in the left ear fluid, genus* Peptostreptococcus* constituted 27% of the flora and genus* Parvimonas *constituted approximately 3% (Figure 1(b)). Similar facultatively anaerobic bacterial species were isolated from concomitant microbiology cultures of the ear fluid samples, while the anaerobic species found by pyrosequencing were not isolated (Table 1). Cultures were done using the four-quadrant semiquantitative method.

**Figure 2:**
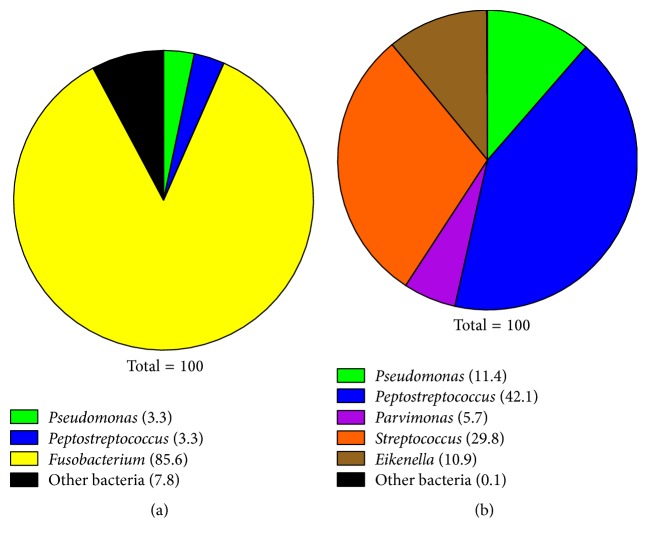
Distribution of major bacterial genera found within biofilms on the TT by deep parallel pyrosequencing. Numbers indicate the percentage of each genus present on the TT from (a) right ear and (b) left ear.* Pseudomonas *and* Streptococcus *are facultative anaerobes;* Fusobacterium, Peptostreptococcus, *and* Parvimonas *are obligate anaerobes;* Eikenella *is a microaerophile (preferring 10% oxygen, 10% carbon dioxide). “Other bacteria” represent genera present at less than 2.5% each. The identities and relative abundance of these organisms are provided in Table 2.

Genera identified from biofilms removed from the TT were similar but found in different proportions. On the TT from the right ear, genus* Fusobacterium *was dominant (86%) followed by the genera* Pseudomonas *and* Streptococcus *at 3% each (Figure 2(a)).* Peptostreptococcus*, at 42%, was the dominant genus found on the TT from the left ear followed by the genera* Streptococcus *(30%),* Pseudomonas *(11%), and* Eikenella *(11%) (Figure 2(b)). Additional genera were present in each specimen at less than 2.5% each. The relative percentages of the species discovered by pyrosequencing, which vary from sample to sample, are listed in Table 2.

### 3.2. *P. aeruginosa* Supports the Growth of* F. nucleatum* under Aerobic Conditions

**Figure 3:**
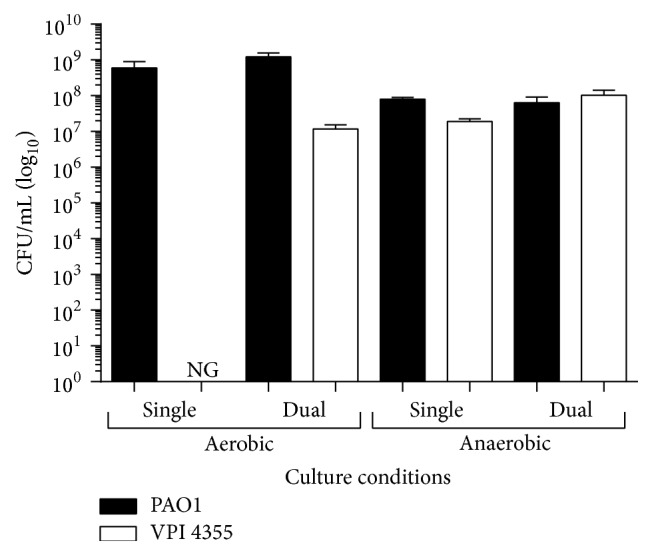
*P. aeruginosa* supports the growth of* F. nucleatum* under aerobic conditions.* P. aeruginosa *strain PAO1 and* F. nucleatum *strain VPI 4355 were inoculated into BHI broth individually or in coculture and incubated at 37°C under aerobic or anaerobic conditions. The number of CFU/mL was determined. Values represent the means of three independent experiments ± SEM.

Previous studies showed that, under aerobic conditions, the growth of aerobic or facultatively anaerobic bacteria within a biofilm reduces the level of oxygen present thereby facilitating the growth of strictly anaerobic bacteria [18]. In patients with PTTO or TT occlusion, pathogenic bacteria within the middle ear may be found in planktonic form within the ear fluid and in biofilms on the mucosal surfaces of the middle ear or on the TT [18]. Our recent pyrosequencing analysis revealed that, within either the left or right ear of the same patient, the ratio of aerobic to anaerobic bacteria within the ear fluid was similar (2.3 : 1 and 2 : 1, resp.) but varied considerably from that on the TT (1.1 : 1 and 1 : 13.6, resp.) (Figures 1 and 2). Additionally, genetic signatures of* P. aeruginosa *and* F. nucleatum *were found in several different patients. Therefore, we examined the potential influence of the facultative bacterium* P. aeruginosa* on the strict anaerobe* F. nucleatum* within this unique environment. We inoculated culture tubes containing BHI broth with the* P. aeruginosa *strain PAO1 and the* F. nucleatum *strain VPI 4355 individually or together (coculture) and incubated the tubes aerobically or anaerobically at 37°C for 24 h. Whether incubated individually or in coculture, PAO1 grew in both environments, although its growth was somewhat less robust when grown anaerobically (Figure 3). As expected, we recovered viable VPI 4355 only from the culture incubated anaerobically when the strain was inoculated individually (Figure 3). However, after 24 h of incubation, the initial inoculum of VPI 4355 in the aerobically incubated coculture had increased by 5 logs and by 6 logs in the anaerobic coculture (Figure 3).

**Figure 4:**
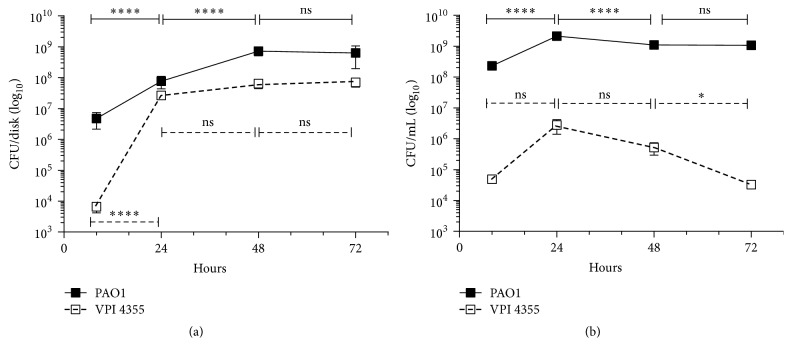
*P. aeruginosa *supports the growth of* F. nucleatum* in both planktonic and biofilm settings under aerobic conditions. Tubes containing 5 mL of BHI broth and a cellulose disk were inoculated with ~10^6^ CFU of* P. aeruginosa* and 10^2^ CFU of* F. nucleatum* and incubated aerobically at 37°C for time indicated on graph. (a) The disks were removed and rinsed to remove planktonic bacteria, and the numbers of CFU in the biofilm (CFU/disk) were determined. (b) The numbers of planktonic bacteria (CFU/mL) were also determined. Values in (a) and (b) represent the means of three independent experiments ± SEM; ^*∗*^*P* < 0.05; ^*∗∗∗∗*^*P* < 0.0001; ns, no significant change.

We then cocultured PAO1 and VPI 4355 aerobically for 72 h and examined for the presence of the bacteria within biofilms formed on cellulose disks and within the planktonic cultures. Over the course of 72 h, PAO1 achieved stationary phase growth within the biofilm (Figure 4(a)). The presence of PAO1 also allowed VPI 4355 to achieve sustained stationary phase growth within the biofilm (Figure 4(a)). Similar to its growth in biofilm, PAO1 achieved stationary phase growth in the planktonic coculture (Figure 4(b)). However, while the CFU of VPI 4355 increased at 24 h, the numbers of CFU declined over the rest of the time course, with a significant drop in CFU between 48 and 72 h (Figure 4(b)). These results suggest that, in an aerobically incubated coculture,* P. aeruginosa *supports the growth of* F. nucleatum* in both the planktonic and biofilm settings, although the biofilm setting appears to support* F. nucleatum *survival and growth better than planktonic culture (Figure 4).

### 3.3. The Growth of* P. aeruginosa* Significantly Reduces the Level of Dissolved Oxygen in Broth

**Figure 5:**
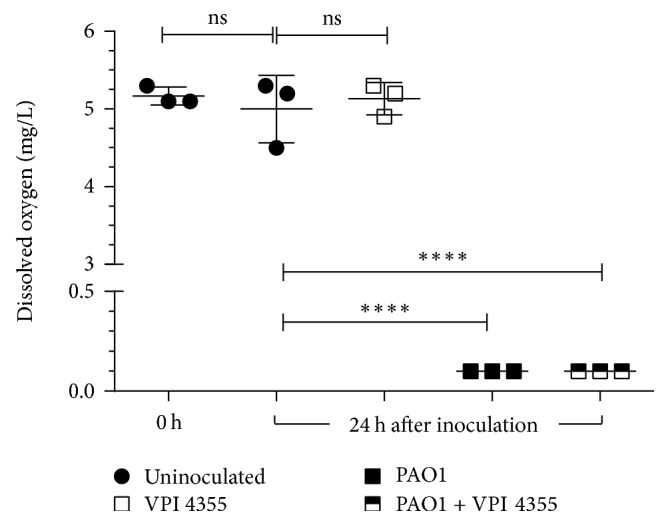
The growth of* P. aeruginosa* significantly reduces the level of dissolved oxygen in broth. Fifty mL conical tubes containing 15 mL of BHI broth were inoculated with ~10^6^ CFU of PAO1, 10^2^ CFU of VPI 4355, or both organisms and incubated aerobically at 37°C. The level of DO_2_ (mg/L) was measured at 24 h after inoculation. As a control, the level of DO_2_ in uninoculated BHI broth was measured at time 0 for a baseline and at 24 h. Values represent the means of three independent experiments ± SEM; ^*∗∗∗∗*^*P* < 0.0001; ns, no significant change.

*P. aeruginosa* may support the growth of* F. nucleatum* by severely restricting the amount of dissolved oxygen (DO_2_) within the medium. To examine this possibility, we inoculated BHI broth with PAO1, VPI 4355, or both, incubated the tubes at 37°C aerobically, and measured the level of DO_2_ at 24 h after inoculation. As a control, we included uninoculated BHI broth. As expected, the level of DO_2_ in the control culture was essentially the same after 24 h of incubation (Figure 5). Similarly, the level of DO_2_ in the individual VPI 4355 culture was comparable to that of the control culture (Figure 5). However, in cultures inoculated with PAO1 alone or in coculture with VPI 4355 the level of DO_2_ was significantly reduced (Figure 5).

**Figure 6:**
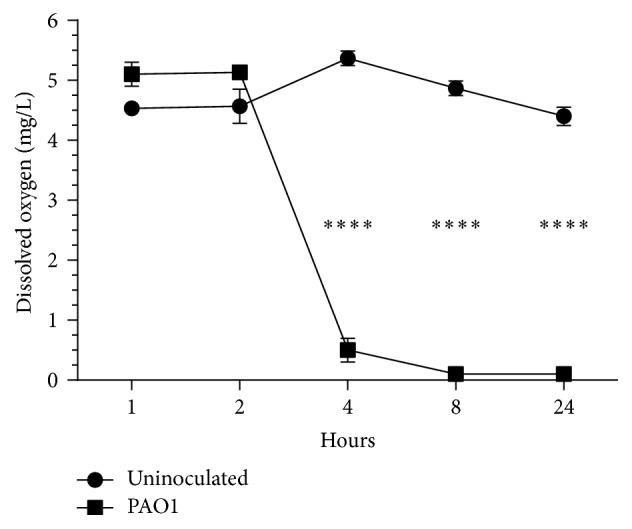
Growth of* P. aeruginosa *creates anaerobic conditions within 4 h of inoculation. Fifty mL tubes of BHI broth were left uninoculated or inoculated with 10^6^ CFU PAO1 and incubated for 24 h at 37°C. Levels of DO_2_ were measured at times indicated. Values represent the means of three independent experiments ± SEM; ^*∗∗∗∗*^*P* < 0.0001.

To determine the time at which the level of DO_2_ in* P. aeruginosa* inoculated cultures falls, we compared the level of DO_2_ in uninoculated BHI broth with the level of DO_2_ in BHI broth inoculated with PAO1. The level of DO_2_ dropped precipitously and significantly by 4 h after inoculation, and by 8 h the level of DO_2_ was essentially zero or anaerobic, an effect which lasted through 24 h (Figure 6). These results suggest that at early stages of growth* P. aeruginosa* reduces the level of DO_2_ sufficiently to sustain the growth of* F. nucleatum*.

### 3.4. Exposure of an Established* P. aeruginosa*/*F. nucleatum* Biofilm to Ambient Oxygen Eliminates* F. nucleatum*

The above results showed that when the environment surrounding the biofilms is anaerobic,* F. nucleatum* survives and grows within a dual-species biofilm. However, it is not known if* F. nucleatum* would survive upon the exposure of the established dual-species biofilm to aerobic conditions, that is, removal from the anoxic conditions within the culture tube. Unlike dental plaque, biofilms that develop on TTs may not be as dense and fluctuation in the oxygen level of the surrounding environment may influence the survival of* F. nucleatum* within the biofilm. To examine these possibilities, we developed two sets of PAO1/VPI 4355 biofilms on cellulose disks in BHI broth as described in Materials and Methods. After 24 h of aerobic incubation (long enough for the broth to become anaerobic), we removed the disks and placed one set on the surface of BHI agar plates and the other set into fresh tubes of BHI broth. Plates and tubes were then incubated aerobically at 37°C for up to 48 h.

**Figure 7:**
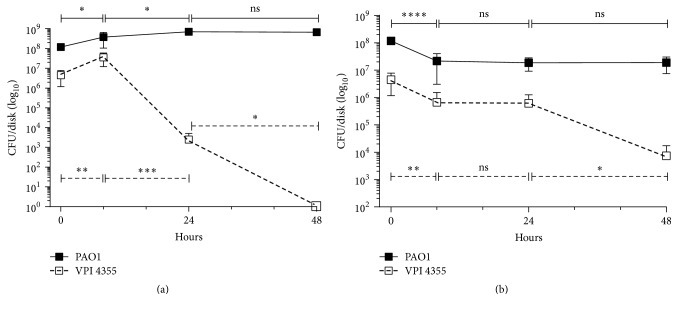
Growth of* F. nucleatum *within the* P. aeruginosa/F. nucleatum *biofilm depends on the environment around the biofilm. PAO1 and VPI 4355 were inoculated into two sets of tubes of BHI broth containing cellulose disks as described in Figure 2 and incubated aerobically for 24 h at 37°C. (a) Disks were removed from one set of tubes, placed on the surface of BHI agar plates, and incubated aerobically for 48 h at 37°C. At intervals indicated on the graph, the disks were removed from the plates and the number of CFU/disk was determined. (b) Disks from the second set of tubes were transferred to fresh tubes of BHI broth and incubated aerobically for 48 h at 37°C. At intervals indicated on the graph, the disks were removed from the broth and the number of CFU/disk was determined. Values in (a) and (b) represent the means of three independent experiments ± SEM; ^*∗*^*P* < 0.05; ^*∗∗*^*P* < 0.01; ^*∗∗∗*^*P* < 0.001; ^*∗∗∗∗*^*P* < 0.0001; ns, no significant change.

*F. nucleatum* within the biofilm transferred to the BHI plate maintained their growth for 8 h after transfer, increasing by ~1 log; but, by 24 h after transfer, the CFU/disk of* F. nucleatum *had declined sharply, and, by 48 h after transfer, no* F. nucleatum *was recovered (Figure 7(a)).* P. aeruginosa, *however, continued to grow throughout the 48 h after transfer (Figure 7(a)). These results suggest that the growth of* P. aeruginosa *within the dual-species biofilm provided* F. nucleatum *with sufficient protection from ambient oxygen to maintain its growth for a short time, but* P. aeruginosa* could not protect against continued exposure to oxygen. The presence of* P. aeruginosa* within the biofilm is essential for the survival of* F. nucleatum*. When we developed a single* F. nucleatum *biofilm in BHI broth anaerobically and transferred the disk to BHI agar that was incubated aerobically, we recovered no CFU as soon as 2 h after placement (data not shown).

In contrast, CFU of both* P. aeruginosa *and* F. nucleatum* within the biofilm transferred to BHI broth decreased by ~1 log in the first 8 h (Figure 7(b)). While the CFU of PAO1 remained stable throughout the next 48 h, the CFU of VPI 4355 remained stable for 24 h and then declined again, dropping to ~10^4^ CFU/disk (Figure 7(b)). These results suggest that the presence of* P. aeruginosa *in the biofilm provided some protection from DO_2_ in the broth. However, it is possible that the limited amount of* P. aeruginosa *released from the biofilm was not sufficient to reduce the DO_2_ within the broth in time to maintain stable growth of* F. nucleatum*. These results suggest that planktonic growth of* Pseudomonas aeruginosa *around a dual-species biofilm is necessary to support the growth of* Fusobacterium nucleatum *within that biofilm.

### 3.5. *F. nucleatum* and* P. aeruginosa* Coaggregate within the Dual-Species Biofilm

**Figure 8:**
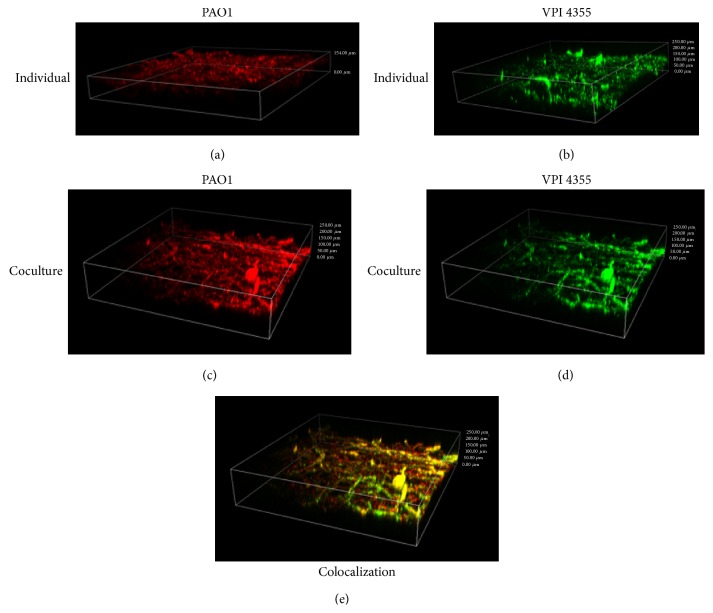
*F. nucleatum* and* P. aeruginosa* coaggregate within the dual-species biofilm. Biofilms of PAO1, VPI 4355, or both were inoculated into BHI broth containing cellulose disks as described in Figure 2. After 24 h, the disks were removed, rinsed, stained by fluorescence in situ hybridization, and examined by confocal laser scanning microscopy. (a) PAO1 alone grown aerobically (red). (b) VPI 4355 alone grown anaerobically (green). (c) PAO1 in dual-species biofilm grown aerobically (red). (d) VPI 4355 in dual-species biofilm grown aerobically (green). (e) Coaggregation of PAO1 and VPI 4355 in dual-species biofilm grown aerobically is indicated by colocalization of red and green stains (yellow). Representative images are shown at 400x magnification.

In mixed-species biofilms, the spatial organization of the different species generally occurs in one of three forms: single-species microcolonies, coaggregates, or layered structures [24]. In the single-species microcolonies, each species forms separate microcolonies while, in the coaggregates, cells of both species intermix [24]. In the layered structure, the species build up in layers, often in a specific order [24]. We examined the organization of the PAO1/VPI 4355 biofilm that was aerobically developed in BHI broth by FISH experiments using a specifically labeled fluorescent probe for each bacterium (Materials and Methods). PAO1 and VPI 4355 developed single-species biofilms under aerobic and anaerobic conditions, respectively (Figure 8). Under aerobic conditions, PAO1/VPI 4355 produced a coaggregated mixed-species biofilm (Figure 8).

## 4. Discussion

As shown in Figure 1 and Table 1, the main microbial pathogen within the ear fluid,* P. aeruginosa*, was identified by both pyrosequencing and the standard laboratory culture. However, in addition to* P. aeruginosa*, pyrosequencing identified other microorganisms including the strict anaerobes,* Peptostreptococcus,* and* Fusobacterium*.* Peptostreptococcus *constituted 27% of the identified genera within the right ear fluid while* Peptostreptococcus* and* Fusobacterium* constituted 10% and 20% of the identified genera within the left ear fluid (Figure 1). The high percentage of these anaerobes suggests that they contribute to the PTTO. Thus, it is possible, as has been demonstrated by their presence affecting the healing process of chronic wounds, that the presence of these anaerobes prevents resolution of PTTO. Pyrosequencing may guide clinicians in facilitating healing of PTTO by defining the mixed populations of microorganisms frequently present in these infections so that appropriate antimicrobial therapy can be instituted. The other intriguing point that pyrosequencing analysis showed is the difference in the composition of the planktonic bacteria within each ear fluid and the bacteria within the biofilms on the TT.

Within a biofilm, the bacterial community is surrounded by an EPS matrix. In a mixed-species biofilm, some pathogens within the biofilm, such as* P. aeruginosa*, may primarily contribute to the EPS, which protects all bacteria within the biofilm, while others, such as* F. nucleatum*, may produce beta-lactamase, protecting all the bacteria within the biofilm from beta-lactam antibiotics [8, 12]. Additionally, certain species within the biofilm may provide the environmental conditions necessary for the survival and growth of other bacterial species. For example, even though the oral cavity is fully aerated, dental plaque (a biofilm) consists of both aerobic and anaerobic bacteria [25]. Studies have shown that* F. nucleatum* serves as a bridge between noncoaggregating early (often aerobes) and late (often anaerobes) colonizers because it is capable of coaggregating with all species of oral bacteria [25, 26]. Perhaps, in this aspect,* F. nucleatum *serves the same role in biofilm formation by otopathogens on TT or on membranes of the middle ear.* P. aeruginosa *and* F. nucleatum *were coaggregated within the PAO1/VPI 4355 biofilm (Figure 8). While we used only two species, our pyrosequencing analysis suggested the presence of many more microbes, both aerobes and anaerobes (data not shown). Similar to the protection from ambient oxygen provided to* F. nucleatum *by the underlying* Streptococcus *species within dental plaque,* P. aeruginosa *provided such protection within our coculture model (Figures 3 and 4). We showed that this protection specifically occurs by the reduction of dissolved oxygen with the fluid culture (Figure 5).

Within an infected site, bacteria interact with each other differently. Some bacteria may support each other's growth by supplying nutritional needs, as has been shown for the oral microflora within the dental plaque [25]. In the present study, the interaction between* P. aeruginosa *and* F. nucleatum *is characterized by* P. aeruginosa* provision of growth support to* F. nucleatum* through reduction of the dissolved oxygen content of the broth medium (Figures 5 and 6). This growth support occurs during planktonic growth but is more evident within the biofilm (Figure 4). Despite the presence of a constant amount of* P. aeruginosa *within the broth, the CFU of planktonic* F. nucleatum *had dropped significantly at 72 h (Figure 4(b)). At this time we do not know the mechanism that led to the reduction of planktonic* F. nucleatum*. The phenomenon is not likely to be caused by the exhaustion of nutrients as* P. aeruginosa* maintained stationary phase growth over the same time period (Figure 4(b)).

In contrast, competitive interactions among bacteria within biofilms also occurs [27]. One species may outcompete others because it can better utilize a given energy source [28]. Other bacteria compete with each other over the limited nutrients by surface blanketing (occupying all the available adhesion sites) or by secreting compounds that actively eliminate other species [29]. Not only does* P. aeruginosa *engage in surface blanketing, it produces extracellular proteins, such as pyocins, that target other competing Gram-negative bacteria including* P. aeruginosa *strains that are sensitive to that specific pyocin [29, 30]. At this time, we cannot exclude the possibility that* P. aeruginosa *produces potential extracellular factors that interfere with the growth of* F. nucleatum*. Using synthetic mucus medium, we have previously shown that* P. aeruginosa *eliminates* S. aureus* from biofilm-like structures within 56 h [31]. These factors may include pyocin, potential quinolone molecules, and other factors. We plan to examine this possibility by aerobically incubating dual BHI cultures of* F. nucleatum *and a* P. aeruginosa *mutant defective in the production of specific factors (e.g., pyocin, quorum sensing molecules) for 72 h and determining if the growth of* F. nucleatum *is maintained.

## 5. Conclusion

**Figure 9:**
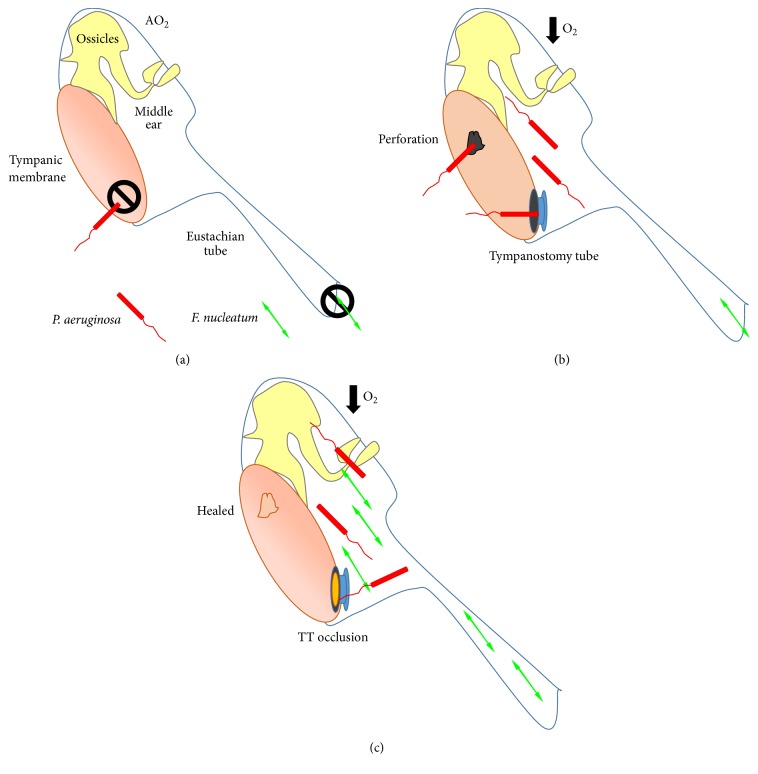
Illustration of the hypothesized mechanism through which the growth of* P. aeruginosa *within the middle ear supports the growth and biofilm development of* F. nucleatum*. The details of the proposed mechanism are described within the text. (a) Noninfected, noninflamed middle ear. (b) Chronic suppurative otitis media (perforation) or chronic serous otitis media with effusion treated with myringotomy and TT insertion allow entry of* P. aeruginosa*, which reduces oxygen with the middle ear.* F. nucleatum *enters middle ear through the Eustachian tube. (c) Perforation heals or TT becomes occluded trapping the organisms within the middle ear where the two bacterial species live together within the middle ear fluid and coaggregate to form biofilms on the TT or on the mucosal surfaces.

In the oropharyngeal cavity, anaerobic bacteria outnumber aerobic bacteria 10–100 : 1 [32, 33]. Environmental and/or ecological changes within the nasopharynx of young children have been reported to favor anaerobic growth [34]. It has been hypothesized that anaerobic bacteria from the oral cavity can travel retrograde up the Eustachian tube into the middle ear cavity, especially when chronic inflammation is present [6]. Chronic suppurative otitis media involves perforation of the tympanic membrane, while the treatment for chronic serous otitis media often involves myringotomy and insertion of TT [7, 12, 15]. Based on our findings, we suggest the following model for the possible interaction between* P. aeruginosa *and* F. nucleatum *and the influence of the environment on their interaction within the middle ear (Figure 9). In the normal ear, the tympanic membrane allows diffusion of oxygen into the middle ear and acts as a barrier to entrance by* P. aeruginosa*, while the noninflamed Eustachian tube prevents entry of oropharyngeal bacteria such as* F. nucleatum *(Figure 9(a)). In the case of a child who suffers a perforated tympanic membrane or has had TT inserted to treat chronic serous otitis media,* P. aeruginosa *gains access to the middle ear (Figure 7(b)). As* P. aeruginosa *grows within the fluid, it significantly reduces the level of the dissolved oxygen and converts the conditions within the fluid into anaerobic conditions (Figure 9(a)).* F. nucleatum *migrates from the oral cavity up the inflamed Eustachian tube into the middle ear cavity where it also becomes established (Figure 9(c)). The two bacterial species live together within the middle ear fluid and coaggregate to form biofilms on the TT or on the mucosal surfaces.

## Figures and Tables

**Table 1:** Bacteria isolated from cultures of left and right ear fluid samples.

Right ear fluid		Left ear fluid	
Organism	SQ^*∗*^	Organism	SQ^*∗*^
*Pseudomonas aeruginosa* strain 1	4+	*Pseudomonas aeruginosa* strain 1	4+
*Pseudomonas aeruginosa *strain 2	4+	*Pseudomonas aeruginosa* strain 2	4+
Viridans streptococci	<1+	Viridans streptococci type 1	<1+
*Staphylococcus* sp. (coagulase-negative)	<1+	Viridans streptococci type 2	<1+
		*Enterococcus faecalis*	<1+
		*Staphylococcus* sp. (coagulase-negative)	<1+
		Diphtheroids	<1+

^*∗*^SQ, semiquantitation based on 4-quadrant streaking method: 4+, growth in quadrant 4; 3+, growth in quadrant 3: 2+, growth in quadrant 2; 1+, growth throughout quadrant 1; <1+, scattered colonies within quadrant 1.

**Table 2:** Relative abundance of genera discovered by pyrosequencing within the ear samples.

Genus^*∗*^	Patient sample			
	Right ear fluid	Right ear TT	Left ear fluid	Left ear TT
*Pseudomonas *	61.8^†^	3.3	66.7	11.4
***Fusobacterium***	20.3	85.6	0.1	0.08
***Peptostreptococcus***	10.2	3.3	27.5	42.1
***Leptotrichia***	2.9	1.7	0.02	0
***Catonella***	1.5	1.4	0.04	0
***Parvimonas***	0.9	0.4	2.8	5.7
***Sneathia***	0.6	0.02	0	0
*Streptococcus *	0.6	0.1	2.1	29.8
***Prevotella***	0.5	1.1	0	0
*Campylobacter*	0.4	1.5	0	0.01
***Porphyromonas***	0.2	0.5	0	0
***Eubacterium***	0.1	0.9	0	0.07
***Sel*** ***e*** ***nomonas***	0	0.04	0	0
*Eikenella*	0	0	0.7	10.9
***Clostridium***	0	0	0.2	0
*Simonsiella*	0	0	0.04	0

^*∗*^Strict anaerobic genera are indicated in bold. ^†^Values indicate percentages of total isolates per sample.
